# Supplementary material for: Impact of mothers’ and fathers’ math self-concept of ability, child-specific beliefs and behaviors on girls’ and boys’ math self-concept of ability
Source: PLoS One. 2025 Feb 12;20(2):e0317837. doi: 10.1371/journal.pone.0317837 (PMC11819543; doi:10.1371/journal.pone.0317837)
Supplement: S1 Table — N = 517, all χ2 values are statistically significant with p < .01. To evaluate ΔCFI, Models 2 and 3 were compared with Model 1. TLI = Tucker-Lewis index, CFI = comparative fit index, RMSEA = root mean square error of approximation, 90% CI = 90% confidence interval RMSEA, SRMR = standardized root mean squared residual. (DOCX) [file pone.0317837.s001.docx]

| **Table S1. Testing Measurement Invariance Across Time and Groups in Measures of Children’s Math Self-Concept of Ability.** | | | | | | | | |
| --- | --- | --- | --- | --- | --- | --- | --- | --- |
|  | χ² | df | TLI | CFI | ∆CFI | RMSEA | 90% CI | SRMR |
| Single-group solutions |  |  |  |  |  |  |  |  |
| Equal form: Girls (*n* = 258) | 115.14 | 72 | .96 | .97 |  | .05 | .03; .06 | .04 |
| Equal Form: Boys (*n* = 259) | 104.97 | 72 | .98 | .97 |  | .04 | .02; .06 | .05 |
| Measurement invariance |  |  |  |  |  |  |  |  |
| Model 1: Equal form | 220.14 | 144 | .96 | .97 |  | .05 | .03; .06 | .05 |
| Model 2: Equal loadings across groups | 250.91 | 159 | .96 | .97 | .00 | .05 | .04; .06 | .10 |
| Model 3: Equal factor loadings across groups and time | 269.17 | 167 | .96 | .97 | .00 | .05 | .04; .06 | .11 |
| *N* = 517, all χ*²* values are statistically significant with *p* < .01. To evaluate ∆CFI, Models 2 and 3 were compared with Model 1. TLI = Tucker-Lewis index, CFI = comparative fit index, RMSEA = root mean square error of approximation, 90 % CI = 90% confidence interval RMSEA, SRMR = standardized root mean squared residual. | | | | | | | | |
