# Supplementary material for: Impact of mothers’ and fathers’ math self-concept of ability, child-specific beliefs and behaviors on girls’ and boys’ math self-concept of ability
Source: PLoS One. 2025 Feb 12;20(2):e0317837. doi: 10.1371/journal.pone.0317837 (PMC11819543; doi:10.1371/journal.pone.0317837)
Supplement: S2 Table — MSC = math self-concept of ability; included, n = 517; excluded, n = 482. *p < .05. ***p < .001 (two-tailed). (DOCX) [file pone.0317837.s002.docx]

**Table S2. Mean Comparisons Included and Excluded Study Participants.**

| Variables | included | | | excluded | | | *t* | *df* | *d* | 95% KI |
| --- | --- | --- | --- | --- | --- | --- | --- | --- | --- | --- |
|  | *M* | *SD* | *n* | *M* | *SD* | *n* |  |  |  |  |
| MSC Child w1 | 5.50 | 1.01 | 517 | 5.37 | 1.05 | 483 | 1.91 | 996 | .12 | <.00; .25 |
| MSC Child w2 | 5.45 | 1.09 | 483 | 5.25 | 1.11 | 396 | 2.52^*^ | 877 | .17 | .04; .30 |
| MSC Child w3 | 4.99 | 1.18 | 400 | 4.84 | 1.18 | 273 | 1.62 | 671 | .13 | -.03; .28 |
| Math Grade w1 | 11.37 | 2.18 | 429 | 10.82 | 2.06 | 409 | 3.73^***^ | 836 | .26 | .12; .39 |

MSC = math self-concept of ability; included, *n* = 517; excluded, *n* = 482.

^*^*p* < .05. ^***^*p* < .001 (two-tailed).
