# Supplementary material for: Impact of mothers’ and fathers’ math self-concept of ability, child-specific beliefs and behaviors on girls’ and boys’ math self-concept of ability
Source: PLoS One. 2025 Feb 12;20(2):e0317837. doi: 10.1371/journal.pone.0317837 (PMC11819543; doi:10.1371/journal.pone.0317837)
Supplement: S3 Table — UNSTD = unstandardized model results, MSC = math self-concept of ability, F/M = mother/father, MSC = math self-concept of ability, M/F = mother/father, grade = math grade w1. aArrow indicates direction of causation. *p < .05. **p < .01. ***p < .001. (DOCX) [file pone.0317837.s003.docx]

| Path^a^ | Father-son | | | Mother-son | | | Father-daughter | | | Mother-daughter | | |
| --- | --- | --- | --- | --- | --- | --- | --- | --- | --- | --- | --- | --- |
|  | UNSTD | SE | STD | UNSTD | SE | STD | UNSTD | SE | STD | UNSTD | SE | STD |
| Education M/F MSC Child w1 | 0.05 | 0.17 | .05 | 0.22 | 0.12 | .13 | 0.12 | 0.15 | .06 | 0.27^*^ | 0.12 | .16^*^ |
| Education M/F MSC Child w2 | 0.00 | 0.04 | .00 | 0.00 | 0.04 | .00 | 0.00 | 0.04 | .00 | 0.10^*^ | 0.04 | .15^*^ |
| Education M/F MSC Child w3 | 0.15^**^ | 0.05 | .15^**^ | 0.02 | 0.06 | .03 | 0.08 | 0.04 | .12 | -0.01 | 0.05 | -.01 |
| Education M/F MSC M/F | 0.42^*^ | 0.19 | .42^*^ | 0.54^**^ | 0.20 | .19^**^ | 0.47 | 0.24 | .17 | 0.45^*^ | 0.18 | .16^*^ |
| Education M/F Expectations M/F | -0.05 | 0.05 | -.09 | 0.05 | 0.04 | .08 | 0.02 | 0.05 | .03 | 0.03 | 0.04 | .04 |
| Education M/F Encouragement M/F | -0.05 | 0.06 | -.07 | -0.06 | 0.06 | -.06 | 0.08 | 0.06 | .10 | -0.01 | 0.09 | .11 |
| Education M/F Grade | 0.56 | 0.30 | .13 | 0.74^**^ | 0.26 | .21^**^ | 0.88^**^ | 0.31 | .21^**^ | 0.76^**^ | 0.26 | .21^**^ |
| Grade MSC Child w1 | 0.70^***^ | 0.18 | .29^***^ | 0.72^***^ | 0.17 | .31^***^ | 0.44^**^ | 0.16 | .19^**^ | 0.40^**^ | 0.15 | .16^*^ |
| Grade MSC Child w2 | 0.09^*^ | 0.04 | .16^*^ | 0.11^**^ | 0.04 | .21^**^ | 0.09 | 0.05 | .16 | 0.10^*^ | 0.05 | .15^*^ |
| Grade MSC Child w3 | 0.07 | 0.06 | .12 | 0.09 | 0.06 | .15 | 0.11^*^ | 0.05 | .20^*^ | -0.01 | 0.05 | -.01 |
| Grade MSC M/F | 0.23 | 0.21 | .08 | 0.52^*^ | 0.26 | .14^*^ | -0.20 | 0.23 | -.07 | 0.56^*^ | 0.28 | .15^*^ |
| Grade Expectations M/F | 0.86^***^ | 0.15 | .41^***^ | 0.86^***^ | 0.15 | .36^***^ | 0.65^**^ | 0.19 | .26^***^ | 0.62^***^ | 0.16 | .27^***^ |
| Grade Encouragement M/F | -0.09 | 0.23 | -.03 | -0.14 | 0.20 | -.04 | -0.17 | 0.25 | -.05 | -0.16 | 0.22 | -.05 |
| UNSTD = unstandardized model results, MSC = math self-concept of ability, F/M = mother/father, MSC = math self-concept of ability, M/F = mother/father, grade = math grade w1.  ^a^Arrow indicates direction of causation.  ^*^*p* < .05. ^**^*p* < .01. ^***^*p* < .001. | | | | | | | | | | | | |

**Table S3. Unstandardized and Standardized Path Coefficients for Stability Coefficients and Covariates (Continuation of Table 3).**
