# Supplementary material for: Impact of mothers’ and fathers’ math self-concept of ability, child-specific beliefs and behaviors on girls’ and boys’ math self-concept of ability
Source: PLoS One. 2025 Feb 12;20(2):e0317837. doi: 10.1371/journal.pone.0317837 (PMC11819543; doi:10.1371/journal.pone.0317837)
Supplement: Attachment — Submitted filename: Response to Reviewers.docx [file pone.0317837.s004.docx]

| Comment | Answer | Excerpt from revised manuscript |
| --- | --- | --- |
| Please ensure that your manuscript meets PLOS ONE's style requirements, including those for file naming. | Thank you for the suggestion, the manuscript has been revised accordingly. |  |
| Please state what role the funders took in the study. If the funders had no role, please state: "The funders had no role in study design, data collection and analysis, decision to publish, or preparation of the manuscript." | The funders had no role in the study and we added this sentence to the resubmission letter. |  |
| Please include your full ethics statement in the ‘Methods’ section of your manuscript file. In your statement, please include the full name of the IRB or ethics committee who approved or waived your study, as well as whether or not you obtained informed written or verbal consent. If consent was waived for your study, please include this information in your statement as well. | We included the following statements in the ‘Method’ section. | Method:  Parents provided written informed consent prior to their children's participation. A letter outlining the study details was sent home with the children, which included an informed consent form for the parents to sign. The signed consent forms were then returned via the children to their teachers. As an incentive for participation, the researchers informed the schools that the population-level results would be shared with them, and that each participating teacher/classroom would receive a small financial contribution, which could be used to purchase educational materials. Data collection was carried out in the classrooms only for those students whose parents had returned the signed consent form. (pp.10-11)  […]  The Ethics Committee of Freie Universität Berlin has confirmed that no ethical approval is required. (p.11) |
| Please include a separate caption for each figure in your manuscript. | Thank you for the suggestion, we included a separate caption for each figure in the manuscript. |  |
| Reviewer 1: Is there any possibility of integrating the school context into the present analyses?  Reviewer 2: This section discusses several key concepts that would require appropriate elaboration. - "“several theories emphasize the importance of parental beliefs and behaviors for the development of school-relevant outcomes in children”: this aspect should be expanded and reference should also be made to other significant adults whose beliefs may have an impact on learning e.g. teachers. | Thanks for the valuable suggestions. We have taken this into account at various points in the ‘Introduction’ and ‘Limitations’ section. | Introduction:  Because parents are considered to be the most important agents of primary socialization [16], several theories emphasize the importance of parental beliefs and behaviors for the development of school-relevant outcomes in children (e.g., [17, 18]). For example, within the *Bioecological Model of Human Development* [17], the immediate social environment, the so-called microsystem, provides a crucial context for human development. Especially in the early years, parents are considered the principal persons within the microsystem; however, over time, interactions with *significant others*—such as teachers and peers—also influence a child’s development. While acknowledging especially the impact of teacher’s beliefs and behaviors on a child’s MSC [19, 20] this paper focuses on parents as the primary socializing agents. (pp. 3-4)  Limitations:  Eighth, as initially mentioned, there are a variety of processes that are involved in the development of ability self-concepts [12, 13]. In order to explore the relative importance of different processes (e.g. interactions with teachers, comparisons with peers and across domains) in the formation of the academic self-concept, follow-up studies should focus on different mechanisms simultaneously. (pp. 31-32) |
| Reviewer 1: In principle, of course, studies on the heritability of academic self-concept would be important - are there already any studies that have investigated the genetic influence and the influence of the environment? If not, this aspect could also be taken up in the discussion. | Thank you for the valuable suggestion. We discussed this point in the ‘Limitations’ section. | Limitations:  Seventh, when studying effects within families, genetic factors must also be considered. Since our study assumed environmental factors within families as mediating processes between parents and child’s MSC, one can also assume that there might be a genetic influence. Twin studies have shown that individual differences in self-perceived abilities can be explained by both genetic factors and non-shared environments [62, 63]. Although the data in this study did not allow us to account for a genetic component, it is important to consider the heritability of academic self-concept when interpreting our findings. (p. 31) |
| Reviewer 1: As the authors stated themselves, it could also be that the results are specific to the recruitment period (up to the 1990s). It is possible that a lot has changed in the last 30 years in terms of parents' self-perceptions of ability and expectations. Maybe the authors could provide more information to validate their results.  Reviewer 2: I have major doubts about this section; the first aspect concerns recruitment and the significant time elapsed since the initial assessment, which for obvious reasons photographs a condition that may not fully represent current functioning both from the point of view of the socio-affective processes involved and the cognitive performance measured. | Thanks for the helpful comments. We have elaborated on this point in the limitations-section and now report stronger empirical evidence to validate our results. | Limitations:  Fourth, it should be noted that the CAB study used data from 1988-1995, which means that the results of this study may not be fully transferable to children the present day. However, the participants in our study are now in their forties, so knowledge of their motivational beliefs during their childhood is still relevant. Furthermore, current studies still reveal gender differences in mathematical ability perceptions of boys and girls [12] and mothers and fathers [60] and parental gender stereotypic beliefs and expectations in math [3, 4, 45]. For example, Starr et al. [45] compared datasets from 1984 to 2009 and found that parents had gender stereotyped beliefs in math across data sets. Furthermore, the importance of parental performance expectations for children’s MSC were also found in a current study [26]. Thus, it is still highly relevant to gain a better understanding of the early mechanism that eventually contribute to these gender difference in math-related beliefs. (pp. 31-32) |
| Reviewer 1: It would have been desirable to provide more than one item to record the parents‘ self-concepts.  Reviewer 2: Another relevant issue relates to the detection of constructs; I find it methodologically flawed to detect parental beliefs by means of a rating item only. This may not seem to capture the conceptual complexity of what is being measured. It is unclear to me why the author(s) did not make use of objective assessment instruments that would certainly have captured the research interest more effectively. This should be justified in the manuscript.  The statistical analyses carried out seem adequate but nevertheless suffer greatly from these arbitrary measurements and therefore risk losing their meaning. The use of this type of measurement should certainly be adequately justified. | We have also responded to this point in the ‘Method’ and ‘Limitations’ section. | Method:  Parents’ child-specific mathematical performance expectations were operationalized using the item “How well do you think this child will do in each of these areas in the next year? Please use this scale: -Math”. Responses could be given on a 7-point Likert response format from *Not at all well* (1) to *Very well* (7). With regard to the criterion validity of the item, correlation patterns with grades are comparable to those found in Pesu et al [26] with multi-item scales (Pesu et al. [26]: *r_mother_* = 41, *r_father_* = 31; in our study: *r_mother_* = 36; *r_father_* = 31, Table S3). (pp. 12-13)  Limitations:  Fifth, parental MSC, their performance expectations and their encouragement were measured with single items. Therefore the reliability of the constructs could not be tested. As stated in the methods section, results of Gogol et al. [47] suggested that single items measures of academic self-concepts meet both validity and reliability criteria. Furthermore, there is evidence that most single-item measures are as valid and reliable as multi-item scales [60]. With regard to performance expectations, we provided additional evidence for criterion validity in the methods section. Moreover, the significant associations of these single items with children’s MSC suggest that these measure meaningful aspects of parental beliefs and practices. (p. 31) |
| Reviewer 1: As the missing values were relatively high for the fathers, it might be helpful to take a look at the analyses without the missings and compare the results of these analyses. Although FIML was used to account for the missing values – I think it could be important to provide additional analyses here in an attachment. | Thank you for this suggestion. We have provided additional information explaining why we believe that FIML is the best approach for handling high percentages of missing data. We also discussed the possibility of providing additional analyses using listwise deletion. We are willing to do this if desired; however, it raises the question of what conclusions should be drawn if, for example, the results differ: “This prohibition should also extend to the intuitive yet misguided practice of using listwise deletion to ‘‘double check’’ the  accuracy of more robust missing data approaches like ML estimation and multiple imputation. There is no good reason for this. When listwise deletion yields discrepant results from ML or MI techniques, this does not in any way cast doubt onto the ML and MI results; rather, it only suggests that  the missingness mechanism is in part MAR (which is quite often the case). Further, there is no logical basis for using listwise deletion in this way. If the listwise result agrees with the ML and MI result, then we will accept the ML and MI result; and if the listwise result disagrees with the ML and MI result, then we will still accept the ML and MI result (because ML and MI provide accurate SEs and are unbiased under both MAR and MCAR mechanisms, whereas listwise deletion provides highly inflated SEs and is only unbiased under MCAR)—the information value of the listwise dele-tion result is nil either way. ” (Newman, 2014, pp. 384-385).  Reference:  Newman DA. Missing data: Five practical guidelines. *Organizational Research Methods*. 2014;17(4):372–411. doi:10.1177/1094428114548590 | Method:  Model parameters were estimated using the FIML algorithm [52]. Studies suggest that FIML outperforms many techniques for handling missing data (e.g. listwise deletion) when the percentage of missing values is relatively high (e.g. 50%; [52, 53]). (p. 15) |
| Reviewer 1: It seems to me that only families with both parents were investigated. What about single parents? Were these parents eliminated from the sample? | As stated in the ‘Method’ section, only children who had at least one parent (mother or father) participating in w1 were included in our study. To avoid any misunderstandings, we provided additional information about the current marital status as reported by mothers. | Method:  Only children who participated in w1 in the study and who had at least one parent (mother or father) participating in w1 were included in our study (*n* = 517). (p. 11)  […]  In w1, 0.6% of mothers stated that they were single, 89.7% were married, 1.7% were separated, 5% were divorced, and 0.6 % were widowed. (p. 12) |
| Reviewer 1: I think it would be helpful to get more information about the sample. For example, for what purpose the families took part in the study and how they were recruited. | Thank you for this comment, we added information of the sample in the ‘Method’ section. | Method:  We draw on data from 517 children and their mothers and fathers from all three cohorts of the CAB Study. CAB is a large-scale longitudinal study that included 10 data collection points, which began in the school year 1986/1987 in four school districts in Southeastern Michigan with three different age cohorts. Recruitment of participants started on December 1st, 1986 and ended October 30th, 1988. Parents provided written informed consent prior to their children's participation. A letter outlining the study details was sent home with the children, which included an informed consent form for the parents to sign. The signed consent forms were then returned via the children to their teachers. As an incentive for participation, the researchers informed the schools that the population-level results would be shared with them, and that each participating teacher/classroom would receive a small financial contribution, which could be used to purchase educational materials. Data collection was carried out in the classrooms only for those students whose parents had returned the signed consent form. (pp. 10-11) |
| Reviewer 1: I have been thinking about whether it would make sense to shed light on the discrepancy in expectations between mothers and fathers. Do you think it could be that the parents view their child differently and this discrepancy is relevant for the MSC of the children? | Thank you for this interesting suggestion. To address the question, whether parents may view their child differently, we added paired *t*-tests to compare mothers’ vs. fathers’ expectations and their encouragement.  How this discrepancy affects childrens’ MSC is also an interesting question. However, we do not think we can answer this point with our research design, as we focus on parent-child dyads (e.g. mothers and daughters) in our study. To address this question, we would have to look at both parents within the same model and possibly form groups according to agreement (e.g. both parents high, only one parent high, both parents low; as in Simpkins et al., 2005). However, we have included this point in the discussion.  Reference:  Simpkins SD, Davis-Kean PE, Eccles JS. Parents' socializing behavior and children's participation in math, science, and computer out-of-school activities. Applied Developmental Science. 2005;9(1):14–30. doi:10.1207/s1532480xads0901_3 | Results:  Additionally, compared to fathers, mothers reported having higher expectations for their daughters’ math performance, *t*(155) = 2.51, *p* = .013, with a small effect size, *d* = .20, 95% CI for *d* [.04, .36]. In contrast, no difference was found between mothers and fathers in how much they encouraged their daughters, *t*(156) = 1.24, *p* = .218. For sons, there were no significant differences between mothers and fathers regarding either their performance expectations, *t*(162) = 0.69, *p* = .493, or their encouragement, *t*(158) = 0.81, *p* = .421. (p. 18)  Limitations:  Ninth, it can be assumed that maternal and paternal socialization do not operate independently, as our model implies. Thus, coordinated parental behavior may have a greater influence on children’s motivational beliefs. Because this study focused on the differential effects of maternal and paternal beliefs and behaviors, it seemed reasonable to consider both parents separately. However, subsequent studies could examine the interaction of both parents, for example, by examining the effects of opposing or coordinated parental beliefs and behaviors [65]. (p. 32) |
| Reviewer 2: With respect to the theoretical framework, the reader is not adequately guided to the theoretical interpretation of the processes involved in learning. This requires a considerable expansion of the specific literature (Parental Socialisation Model).  "children seem to adopt their parents’ interpretations of reality and integrate them into their own self-related cognitions": this part is very interesting it needs to be expanded and justified with scientific studies. | We added some more information about the model and the learning mechanisms in the specific sections about the model components. | Introduction:  Within the *Parental Socialization Model*, parents’ general beliefs are assumed to affect children’s motivation directly through different mechanisms: First, in line with the social learning theory [16], parents act as role models. Since children are most likely to act like people who are similar to them [22], e.g., same-sex parents, it is thought that gender differences in children’s motivation are a consequence of differential behaviors (i.e. role modeling) of mothers and fathers [21]. Second, parents may communicate their self-beliefs more directly to their children. By incorporating the messages mothers and fathers provide about their own self-perceptions, boys and girls may develop differential self-beliefs [21]. (pp. 4-5)  […]  In addition to the direct links between parents' general beliefs and children's motivation, the Parental Socialization Model [21] suggests an additional, indirect link through parents' beliefs about their child, such as performance expectations. Specifically, the model posits that parents' general beliefs—such as their own MSC—influence what they expect from their children. These expectations, in turn, shape their children's MSC. Regarding the link between parents’ expectations and the MSC of their children, two mechanisms are expected: First, parents may provide varied learning opportunities based on their beliefs about their child’s abilities, such as domain-specific encouragement (as discussed in the following section). Second, parents often communicate these beliefs directly through messages about their child’s abilities, helping the child interpret their own experiences. As a result, children seem to adopt their parents’ interpretations of reality and integrate them into their own self-related cognitions [21]. (pp. 5-6) |
| Reviewer 2: “extensive research has shown that there is a positive relation between mothers’ and fathers’ child-specific math performance expectations and children’s MSC for different samples with respect to age and nationality”: this expression is not enough, some more relevant work on this topic is needed. | Thank you for this suggestion. We expanded this section as described in the right column. | Introduction:  In recent decades, extensive research has shown that there is a positive relation between mothers’ and fathers’ child-specific math performance expectations and children’s MSC for different samples with respect to age and nationality [12, 17, 23, 25, 26, 27, 28]. For instance, Pesu et al. [28] found in a Finnish sample that mothers’ and fathers’ beliefs about their child’s abilities in 7th grade predicted adolescents’ mathematics self-concept (MSC) in 9th grade, even after controlling for previous MSC and mathematics performance. Similarly, Tiedemann [28] found in a German sample of 3rd and 4th graders that, independent of prior math grades and teachers’ perceptions of ability, parents’ beliefs about their child’s abilities in math predicted the child’s MSC. In a US sample, Gniewosz et al. [29] found, that the relation between mothers' beliefs about their child's mathematics competence and the child’s MSC increased during the transition to secondary school, while the effect of grades decreased. Unlike grades, parental performance expectations remained more stable across time, leading the authors to conclude that students perceive parental competence beliefs as more reliable, thus placing greater emphasis on them [29]. (p. 6) |
| Reviewer 2: “there is evidence that parents’ math anxiety is transferred to their children, with stronger effects in same-sex dyads”: this sentence is very disconnected from the rest and yet it is not even deepened; it is necessary to avoid these situations by expanding and rewriting the entire period. | Thank you for the comment, we revised the whole section ‘General Beliefs: Mothers’ and Fathers’ MSC’. | Introduction:  Within the *Parental Socialization Model*, parents’ general beliefs are assumed to affect children’s motivation directly through different mechanisms: First, in line with the social learning theory [16], parents act as role models. Since children are most likely to act like people who are similar to them [22], e.g., same-sex parents, it is thought that gender differences in children’s motivation are a consequence of differential behaviors (i.e. role modeling) of mothers and fathers [21]. Second, parents may communicate their self-beliefs more directly to their children. By incorporating the messages mothers and fathers provide about their own self-perceptions, boys and girls may develop differential self-beliefs [21]. Taking into account that both girls and mothers have lower MSC than boys and fathers, respectively (e.g., [3, 6, 23]), one could assume that there is a gendered intergenerational transmission of paternal and maternal beliefs regarding mathematics. By parents passing on their own self-beliefs directly or through exemplary behavior, children might learn what behaviors and beliefs are considered appropriate according to their gender roles, making it more likely that girls will adopt their mothers’ beliefs and boys their fathers’ beliefs [23].  However, despite these theoretically assumed mechanisms, the relation between mothers’/fathers’ and girls’/boys’ MSC has received little attention in research. To our knowledge, only two studies explored the direct link, with somewhat mixed findings. Eccles- Parsons et al. [23] found only weak evidence for a relation between mothers’ and fathers’ MSC and girls’ and boys’ MSC from fifth to eleventh grade. Theurer et al. [24] found that parental MSC at the end of second grade longitudinally predicted children’s MSC one year later. In a separate gender analysis, only girls’ MSC were predicted by their parents’ MSC. Because the questionnaires were largely completed by the mothers (75% mothers, 20% both parents, 7% fathers), the authors [24] concluded that these relations might be stronger in same-sex dyads. (pp. 4-5) |
| Reviewer 2: Finally, the introduction does not make explicit the ‘gap’ in the literature that the study will fill. Is it the evolution over the life span? The implications in reference to performance? It might prove useful to better integrate this aspect with the results already available in the literature. | Thank you for the comment. All references to research gaps have been inserted in the right-hand column. | Introduction:  In educational psychology research, studies have primarily focused on mothers or composite measure combining mothers and fathers. Therefore, little is known about whether frameworks for parental socialization work similarly for fathers and mothers. To close this gap, drawing on data of the Childhood and Beyond Study [15], this study investigates the indirect effects between mothers’ and fathers’ MSC and their children’s MSC via parents’ performance expectations and math- and science-related encouragement, and the differential relations for boys and girls. (p. 3)  […]  However, despite these theoretically assumed mechanisms, the relation between mothers’/fathers’ and girls’/boys’ MSC has received little attention in research. (p. 5)  […]  Given the empirical evidence suggesting that parental expectations shape children’s MSC, it is crucial to investigate the factors that might affect parental expectations, like parents’ own MSC. For example, it could be assumed that parents may generalize their own beliefs into their expectations of their child’s performance. Research findings on the relations between parents’ own self-concept of ability and their child-specific performance expectations are scarce. (p. 7)  […]  Although this pathway is theorized, the link between mothers’ and fathers’ own MSC and their math-specific encouragement has not been investigated yet. (p. 8)  […]  Largely unresolved is the question of the extent to which maternal and paternal encouragement of domain-specific activities differentially impact daughters and sons. For example, four of the studies cited above did not distinguish between parental gender [37-40]. Since the samples used were predominantly mothers, previous research largely illustrates how maternal behaviors influence children’s motivational beliefs. The work by Simpkins et al. [27] took the differential impact of fathers and mothers into account. Here, they found that paternal behaviors had an impact on children’s MSC in elementary school, but maternal behaviors did not. However, it remains unclear, how paternal and maternal behaviors affect boys’ and girls’ MSC beyond elementary school. (pp. 8-9) |
| Reviewer 2: The conclusions are not appropriate for the scientific product; this section should be completely revised by expanding the relations between the constructs in light of the results obtained. The entire dimension related to the results of the study and the findings should also be stressed. | Thank you for the suggestion. We revised the ‘Conclusion’ section. | Conclusion:  Studies suggest that girls hold a lower math self-concept of ability (MSC) compared to boys [3-6]. These gender differences were thought to contribute to disparities in students' educational and career choices [7-11]. This suggests a need to examine the mechanisms that contribute to children’s MSC. Based on the Parental Socialization Model, our study expands the prior literature by testing the links between mothers’ and fathers’ own MSC, their child-specific performance expectations, and their encouragement regarding math and science activities in elementary school on boys’ and girls’ MSC in both elementary and secondary school. While we found no gender differences in parental expectations and encouragement of girls and boys, we found different mechanisms that contribute to their MSCs.  Overall, the results of this study suggest that mothers and fathers tended to translate their own MSC into their mathematical performance expectations and encouragement of their sons, but not their daughters. This mechanism could contribute to the maintenance of gender differences, as parental expectations, especially father’s performance expectations, have been shown to have an important effect on children's MSC. These findings suggest that frameworks for parental socialization do not apply in the same way for boys and girls and that a differentiation according to child’s gender is highly relevant and should therefore be considered in follow-up studies.  Furthermore, we investigated the role of parents own MSC. Our results suggest that only fathers’ MSC was indirectly related to sons’ MSC via performance expectations. This suggests that fathers who view themselves as capable in mathematics are more likely to expect similar abilities in their sons, thereby fostering their sons' confidence in math. Contrary to our hypothesis, however, we found that the effects of fathers’ MSC, compared to the effects of mothers MSC, on sons’ MSC were not significantly different. This means that although we find an indirect effect of the fathers’ MSC on the sons’ MSC, this effect is not significantly greater than in the mother-son dyad. Considering the small sample sizes in the dyads, this could be due to the low power of the study, which is why this hypothesis should be tested in follow-up studies with larger samples. Furthermore, both boys’ and girls’ MSC were predicted by their fathers’ performance expectations and girls’ MSC by their fathers’ math-specific encouragement.  Our findings highlight the relevance of fathers’ early educational encouragement of math and science activities and may lead to effective interventions to improve boys’ and girls’ mathematical competence beliefs. The mere knowledge of what role fathers play in the development of their children’ MSC, could result in a change in their beliefs and behaviors. As studies show, that fathers’ school- and home-based involvement is lower than mothers’ (for an overview see [58]), interventions should raise awareness among fathers and seek to increase their educational participation. Future studies should build on our findings and investigate gendered parent-child mechanisms in other subjects and domains. Furthermore, to place our findings in a larger picture, different parental behaviors should be taken into account. (pp. 32-34) |
